# Supplementary material for: Oligotrophic wetland sediments susceptible to shifts in microbiomes and mercury cycling with dissolved organic matter addition
Source: PeerJ. 2018 Apr 3;6:e4575. doi: 10.7717/peerj.4575 (PMC5888151; doi:10.7717/peerj.4575)
Supplement: Supplemental Information 1 [file peerj-06-4575-s001.docx]

**SUPPLEMENTAL MATERIAL**

**Material and Methods.**

*Experimental design.*

A block sampling design was employed in which samples for replicates of both treatments were obtained in one of five randomly chosen 0.5 x 0.5-m blocks at each site. Nalgene bottles were capped within the sediment and immediately placed in two Mylar bags with oxygen absorbing packets to maintain anoxic conditions. Sediment samples were transported on ice to the University of Colorado at Boulder, stored at 4^o^C for a total of 8 days, and homogenized on a shaker table at 250 RPM for 2 hours before incubation construction. Plant matter was air dried for 24 hours, then transported to CU- Boulder, and oven dried at 60^o^C for 24 hours to prevent mold growth during experiment set up. Dried plant matter was ground, and 1g of dry plant material was leached in 20 mL of Nanopure water on a shaker table at 250 rpm for one hour and at rest for four hours. Leachate was then filtered through Whatman 0.7 μm glass microfiber filters (Whatman Incorporated, Florham Park, NJ, USA) that had been burned at 450^o^C for four hours to remove residual carbon.

*Sediment chemistry and extracellular enzyme activity.*

Percent carbon and nitrogen were determined on 25 mg of dried sediment (100^o^C for 48 hours) packed in tin capsules and analyzed on a Thermo Finnigan EA 1112 Series Flash Elemental Analyzer (Thermo Fisher Scientific, Inc., Waltham, MA, USA)(Matejovic 1997). Within a day of the incubation start, ~10 g fresh sediment for each replicate was extracted in 40 mL 0.5M K_2_SO_4_ to determine initial sediment NH_4_^+^ and NO_3_^−^/NO_2_^−^ extractable N, total non-purgeable organic carbon (NPOC), and total dissolved nitrogen (TDN). All extractions included shaking for one hour and filtering with Whatman no.1 paper (Whatman Incorporated, Florham Park, NJ, USA). Extracts were frozen until chemical analysis. NH_4_^+^ was measured on a Lachat QuikChem 8500 Flow Injection Analyzer (Lachat Instruments, Hach Company, Loveland, CO, USA) and NO_3_^−^/NO_2_^−^ were measured on a BioTek Synergy 2 Multidetection Microplate Reader (BioTek, Winooski, VT, USA). NPOC was measured on a Shimadzu TOC-V CSN Total Organic Carbon Analyzer (Shimadzu TOCvcpn, Kyoto, Japan). Sediment pH was determined on dried sediment using a ratio of 2 g sediment to 4 mL DI H2O according to standard methods(Nemergut et al. 2007). Extracellular enzyme activities of β-1,4-glucosidase (BG), β -1,4-N-acetylglucosaminidase (NAG), and acid phosphatase (aP) were determined from subsamples at the beginning and end of the incubation via fluorometric microplate methods(Saiya-Cork et al. 2002; Sinsabaugh et al. 2002; Weintraub et al. 2013) according to the protocol described in Weintraub *et al.*^5^ using ~2g fresh sediment and sodium acetate buffer with a pH of 6.0 to assess rates of biological activity.

*Dissolved organic matter characterization.*

Non-purgeable organic carbon (NPOC) was measured on a Shimadzu TOC-V CSN Total Organic Carbon Analyzer (Shimadzu TOCvcpn, Kyoto, Japan), and dissolved organic matter characteristics were determined using fluorescence spectroscopy to analyze the excitation-emission spectra (EEM) of optically active DOM pools. UV–vis analysis was performed using an Agilent 8453 spectrophotometer (Agilent Technologies, Santa Clara, CA, USA), and samples were diluted with DI H_2_O such that UV absorbance at 254 nm fell between 0.1 and 0.2 cm^−1^(Miller & McKnight 2010). A Fluoromax-3 spectrofluorometer (Horiba Jobin Yvon, Kyoto, Japan) was used to collect an EEM for each sample at excitation wavelengths of 245-450 nm in increments of 10 nm, and at emission wavelengths of 300-600 nm in increments of 2 nm and EEMs were corrected for instrument biases(Cory et al. 2010).

*Microbial DNA extraction and sequencing.*

After extraction, DNA samples were eluted and stored in TE buffer until sequencing preparation. The region encoding the V4 fragment of the 16S rRNA gene was amplified with the primers 515F/806R, using the PCR protocol described by the Earth Microbiome Project(Caporaso et al. 2012). The forward primer included the 5’ illumina adaptor, a forward primer pad, and forward primer linker followed by the forward 515 primer. The reverse primer contained the reverse complement of the 3’ adaptor, golay barcode, reverse primer pad, reverse primer linker, and the reverse 806 primer. PCR was run with a reaction of 25.0 μL including 8.6 μL PCR Grade H2O (Teknova Catalog #W3350), 12.5 μL FideliTaq Master Mix (Affymetrix Catalog # 71182), 1.0 μL Primer 515F (10μM), 1.0 μL Primer 806R (10μM), 0.11 μL MgCl_2_ (25mM) in order to quench EDTA, and 2.79 μL Template DNA (all samples normalized to 3.583ng/μL). Samples and negative controls were denatured at 94°C for 2 minutes and then amplified in 25 cycles at 94°C for 45 seconds, 50°C for 60 seconds, and 68°C for 45 seconds. A final extension was included of 5 minutes at 68°C. All PCR products were run in triplicate and then combined into single samples. To eliminate primer dimer contamination, barcoded PCR product was purified using the QIAquick Gel Extraction Kit (Qiagen, Cat. # 28704), according to the manufacturer’s protocol. Samples were multiplexed according to DNA concentration as per quantification using the PicoGreen method (Invitrogen, Cat. # P11496) on a microplate reader according to the manufacturer’s protocol. Pooled DNA purity and quality was determined on a NanoDrop800. Final pooled DNA was purified using the UltraClean PCR Clean-up Kit, according to the manufacturer’s protocol.

Illumina sequences of partial 16S rRNA gene were filtered for sequence length and minimum quality score in the UPARSE pipeline(Edgar 2013). After chimera checking, Operational Taxanomic Units (OTUs) were determined using a *de novo* approach with a 97% sequence similarity threshold using default parameters and workflow for joined, paired-end reads in UPARSE. QIIME(Caporaso et al. 2010) was utilized for downstream sequence analysis, and sequences were rarefied to the lowest number of sequences in a sample. Sequences were aligned and used to build a phylogenetic tree, as described by Knelman *et al.*(2014) using the ‘clearcut’ algorithm.

For overall community structure, a total of 2,178,342 sequences were recovered (average 22,691.06 per sample) with a minimum of 10,664 and a maximum of 71,457 per sample, and samples were rarefied to 10,664 sequences per sample. For methylators, a total of 25,222 sequences matching our methylator database were recovered (average 262.73 per sample) with a minimum of 107 and a maximum of 786 per sample, and samples were rarefied to 107 sequences per sample.

**Dissolved organic matter supplement.**

We examined DOM concentration and characteristics to infer the extent to which DOM measurements in microcosm water was representative of sediment DOM processing. In brief, DOM characteristics in both environments were consistent with sediment-to-water DOM flux, either through physical exchange of ambient organic carbon across the sediment-water interface or through the production and diffusion of organic carbon by sediment microorganisms. DOM characteristics at day 7 differed between leachate and no leachate microcosms for both environments, and demonstrating leachate microcosms were expectedly influenced by DOM addition. Further, underlying DOM processing mechanisms are not drastically altered by leachate addition and that these mechanisms may vary across a vegetation gradient, as trends of DOM characteristics through time were consistent within each environment regardless of leachate addition.

For each microcosm, NPOC concentration, DOM fluorescence intensity, and DOM fluorescence indices were measured in the applied leachate (if any) and at each time point (days 0, 7, 14, 21, and 28). In both environment types, leachate microcosms displayed an initial decrease in water NPOC content between leachate application at day 0 and day 7, a trend that continued between days 7 and 28 within vegetated but not unvegetated microcosms (Figure S3A). The initial decrease in NPOC concentration suggests a net downward flux of C at the sediment-water interface due to sediment sorption and/or microbial processing of NPOC in the water column in response to leachate addition. Vegetated microcosms displayed higher rates of biological activity (using extracellular enzyme activity as a proxy). Labile organic matter can serve as a readily available carbon source for a wide range of organisms(Berggren et al. 2010; Keil & Kirchman 1991); thus, addition of plant leachate may have induced the rapid degradation of organic matter. Vegetated environments may have been more predisposed for higher rates of NPOC processing by containing a larger proportion of heterotrophic bacteria than unvegetated environments. Because this downward trend in NPOC concentrations continued after day 7 in vegetated but not unvegetated microcosms, these processes may be of greater importance in vegetated environments that was more eutrophic and displayed higher rates of *in situ* biological activity.

In contrast, sediments not receiving leachate experienced an increase in water NPOC, putatively do to POC desorption and/or microbial processing within sediments. Control microcosms for both environment types increased between day 14 and day 28 (Figure S3B). Some of this observed increase is undoubtedly due to leaching of ambient sediment C into water, as microcosms were amended with a relatively small volume of water (250 mL) relative to sediment mass (50 g), and small amounts of leached carbon would have generated large increases in water NPOC. Alternatively, increases in NPOC could result from chemolithotrophic organisms (*e.g.*, sulfate-reducing, iron-reducing, and methanonic organisms) or chemoorgantrophic organisms that are prevalent within anoxic environments and can produce dissolved organic carbon as a product of their metabolisms. Microcosms without leachate were not subject to artificial selective pressures for heterotrophy (imposed by leachate addition), and these microcosms have maintained chemolithic microbial communities to a greater extent that those receiving leachate. Additionally, leachate may mitigate inorganic mercury toxicity to heterotrophs through physical interaction, and a decrease in DOM may be due to metal sequestration in sediments via geochemical mechanisms(Drexel et al. 2002; Haitzer et al. 2002; Skyllberg et al. 2000). Finally, the proportion of fluorescent DOM was stable in all microcosms, indicating that observed fluorophores observed were more reflective of DOM released from sediments than of changes in DOM in the water column.

The fluorescence intensity of DOM did not vary across time within any set of microcosms (Figure S3C and S3D, veg. no leachate (across days 7, 14, 21, and 28): *P =* 0.08, *R^2^* = 0.21, veg. leachate (across days 0, 7, 14, 21, and 28): *P =* 0.65, unveg. no leachate (across days 7, 14, 21, and 28): *P =* 0.45, unveg. leachate (across days 0, 7, 14, 21, and 28): *P =* 0.79), and the ratio of fluorescence intensity (SUVA_254_) to NPOC content (fluor:NPOC)(Figure S1E and S1F, veg. no leachate (across days 7, 14, 21, and 28): *P =* 0.11, veg. leachate (across days 0, 7, 14, 21, and 28): *P =* 0.27, unveg. no leachate: *P =* 0.02, *R^2^* = 0.29, unveg. leachate (across days 0, 7, 14, 21, and 28): *P =* 0.25) was stable in all microcosms except unvegetated microcosms without leachate, indicating that this set may have leached a greater proportion of non-fluorescent C from sediment than other microcosms.

Importantly, DOM indices for both leachate and no leachate followed similar trends in each environment, indicating that changes in fluorescence indices within all microcosms are likely reflective of the diffusion of fluorescent material from sediments. No leachate microcrosms received no DOM inputs at day 0, and thus, all DOM must be representative of diffused sediment organic matter, consistent with our results (Figure S3B). Though it is possible fluorescence indices in leachate microcosms denote some processing of DOM external to sediment processes, if fluorescence in leachate microcosms were more reflective of DOM processing within water NPOC, one would not necessarily expect to observe the same trends in leachate as in no leachate microcosms. Moreover, as FI increased through time in most microcosms (main text, Figure 4) while fluor:NPOC remained mostly stable, fluorescent DOM appeared to be influenced by the addition of microbially-derived DOM to the NPOC pool, although it is also possible that a portion of this DOM was generated before the onset of our incubation.

The processing of DOM may have also varied temporally during our incubation period, as vegetated environment microcosms showed more pronounced changes in fluorescence indices between day 14 and day 28 than day 0 and day 14 in both leachate and no leachate groups, with the exception of HIX in leachate microcosms (main text, Figure 4). As this effect occurred around day 14, it may have been due to substrate limitation for metabolisms reliant on chemical species that were not added to our incubations. For example, sulfate- and iron-reducing bacteria can be major contributors to C cycling within anoxic sediments, and the limitation of these processes may have reduced the contribution of chemolithotrophs to the fluorescent DOM pool. By contrast, only trends in FI in unvegetated environment microcosms appeared to differ between day 0 and day 14 vs. day 14 and day 28, with a more drastic increase in FI during the day 14 – day 28 period. It is possible that DOM processing in unvegetated sediments is more representative of chemoorganotrophs that are not dependent on inorganic substrate for energy generation. The increase in FI between day 14 and day 28 is also coincident with a trend for increasing water NPOC content during this period in both leachate and no leachate microcosms, possibly indicative of increased production and/or diffusion of microbially-derived DOM from sediments during this time interval.

As a whole, our data suggest that the trends in DOM fluorescence indices and the correlations between microbial community structure and HIX presented in the main text are reflective of microbial processing of fluorescent organic material within sediments.

**Literature Cited.**

Berggren M, Laudon H, Haei M, Ström L, and Jansson M. 2010. Efficient aquatic bacterial metabolism of dissolved low-molecular-weight compounds from terrestrial sources. *The ISME journal* 4:408-416.

Caporaso JG, Kuczynski J, Stombaugh J, Bittinger K, Bushman FD, Costello EK, Fierer N, Pena AG, Goodrich JK, and Gordon JI. 2010. QIIME allows analysis of high-throughput community sequencing data. *Nature methods* 7:335-336.

Caporaso JG, Lauber CL, Walters WA, Berg-Lyons D, Huntley J, Fierer N, Owens SM, Betley J, Fraser L, and Bauer M. 2012. Ultra-high-throughput microbial community analysis on the Illumina HiSeq and MiSeq platforms. *The ISME journal* 6:1621-1624.

Cory RM, Miller MP, McKnight DM, Guerard JJ, and Miller PL. 2010. Effect of instrument‐specific response on the analysis of fulvic acid fluorescence spectra. *Limnology and Oceanography: Methods* 8:67-78.

Drexel RT, Haitzer M, Ryan JN, Aiken GR, and Nagy KL. 2002. Mercury (II) sorption to two Florida Everglades peats: Evidence for strong and weak binding and competition by dissolved organic matter released from the peat. *Environmental Science & Technology* 36:4058-4064.

Edgar RC. 2013. UPARSE: highly accurate OTU sequences from microbial amplicon reads. *Nature methods* 10:996-998.

Haitzer M, Aiken GR, and Ryan JN. 2002. Binding of mercury (II) to dissolved organic matter: the role of the mercury-to-DOM concentration ratio. *Environmental Science & Technology* 36:3564-3570.

Keil RG, and Kirchman DL. 1991. Dissolved combined amino acids in marine waters as determined by a vapor-phase hydrolysis method. *Marine Chemistry* 33:243-259.

Knelman JE, Schmidt SK, Lynch RC, Darcy JL, Castle SC, Cleveland CC, and Nemergut DR. 2014. Nutrient Addition Dramatically Accelerates Microbial Community Succession. *PloS one* 9:e102609.

Matejovic I. 1997. Determination of carbon and nitrogen in samples of various soils by the dry combustion. *Communications in Soil Science & Plant Analysis* 28:1499-1511.

Miller MP, and McKnight DM. 2010. Comparison of seasonal changes in fluorescent dissolved organic matter among aquatic lake and stream sites in the Green Lakes Valley. *Journal of Geophysical Research: Biogeosciences (2005–2012)* 115.

Nemergut DR, Anderson SP, Cleveland CC, Martin AP, Miller AE, Seimon A, and Schmidt SK. 2007. Microbial community succession in an unvegetated, recently deglaciated soil. *Microbial Ecology* 53:110-122.

Saiya-Cork K, Sinsabaugh R, and Zak D. 2002. The effects of long term nitrogen deposition on extracellular enzyme activity in an Acer saccharum forest soil. *Soil Biology and Biochemistry* 34:1309-1315.

Sinsabaugh R, Carreiro M, and Repert D. 2002. Allocation of extracellular enzymatic activity in relation to litter composition, N deposition, and mass loss. *Biogeochemistry* 60:1-24.

Skyllberg U, Xia K, Bloom PR, Nater EA, and Bleam WF. 2000. Binding of mercury (II) to reduced sulfur in soil organic matter along upland-peat soil transects. *Journal of environmental quality* 29:855-865.

Weintraub SR, Wieder WR, Cleveland CC, and Townsend AR. 2013. Organic matter inputs shift soil enzyme activity and allocation patterns in a wet tropical forest. *Biogeochemistry* 114:313-326.

**Figures.**

**Figure S1.** Changes in microbial community structure (across days 0, 7, 14, 21, and 28) were assessed with ANOSIM and visualized with NMDS. (A) Vegetated and (B) unvegetated microcosms are denoted in Figure S2. Shifts were greatest among unvegetated microcosms without leachate addition. Colors represent sampling time points, and treatments are denoted by closed triangles and open circles

**Figure S2.** Partial and complete 16S rRNA sequences from the NCBI GenBank database for all putative methylators were used to construct a phylogenetic tree using the ‘clearcut’ algorithm in QIIME. The tree is rooted at its midpoint, and organisms present in our samples are denoted in blue, with OTUs in the family *Peptococcaceae* (which was more abundant when DOM was added to unvegetated sediments) are colored red. The length of the bar represents 5% sequence divergence

**Figure S3.** DOM properties were assessed through time with linear and quadratic regressions in each environment and treatment. Averages for each environment and treatment are plotted at days 0, 7, 14, 21, and 28, with error bars representing the standard error. Points at day 0 represent the properties of the applied leachate. Plots in the first column are leachate microcosms, while plots in the second column are no leachate microcosms. Unvegetated microcosms are depicted as closed circles with dashed lines showing significant regressions; vegetated microcosms are x’s with solid lines showing significant regressions. (A) depicts changes in NPOC in leachate microcosms, while (B) denotes the same changes in no leachate microcosms. (C) shows changes in fluorescence (SUVA_254_) in leachate microcosms, and (D) denotes the same changes in no leachate microcosms Lastly, (E and F) show changes in SUVA_254_ normalized by DOC in leachate and no leachate microcosms, respectively.

**Tables.**

**Table S1.** OTUs identified by SIMPER analysis on the full community between day 0 and day 28 in unvegetated leachate microcosms are listed in Table S1.

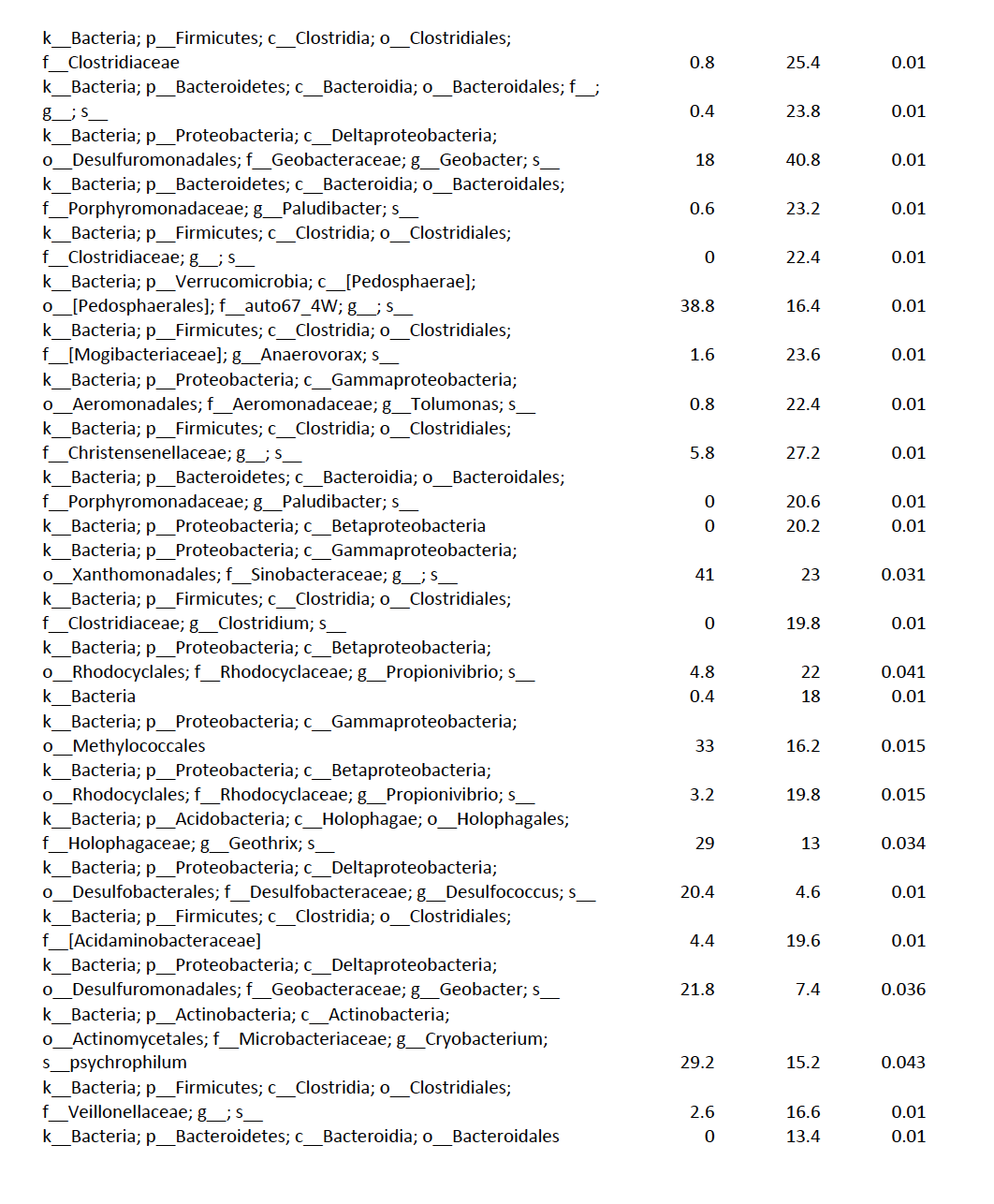

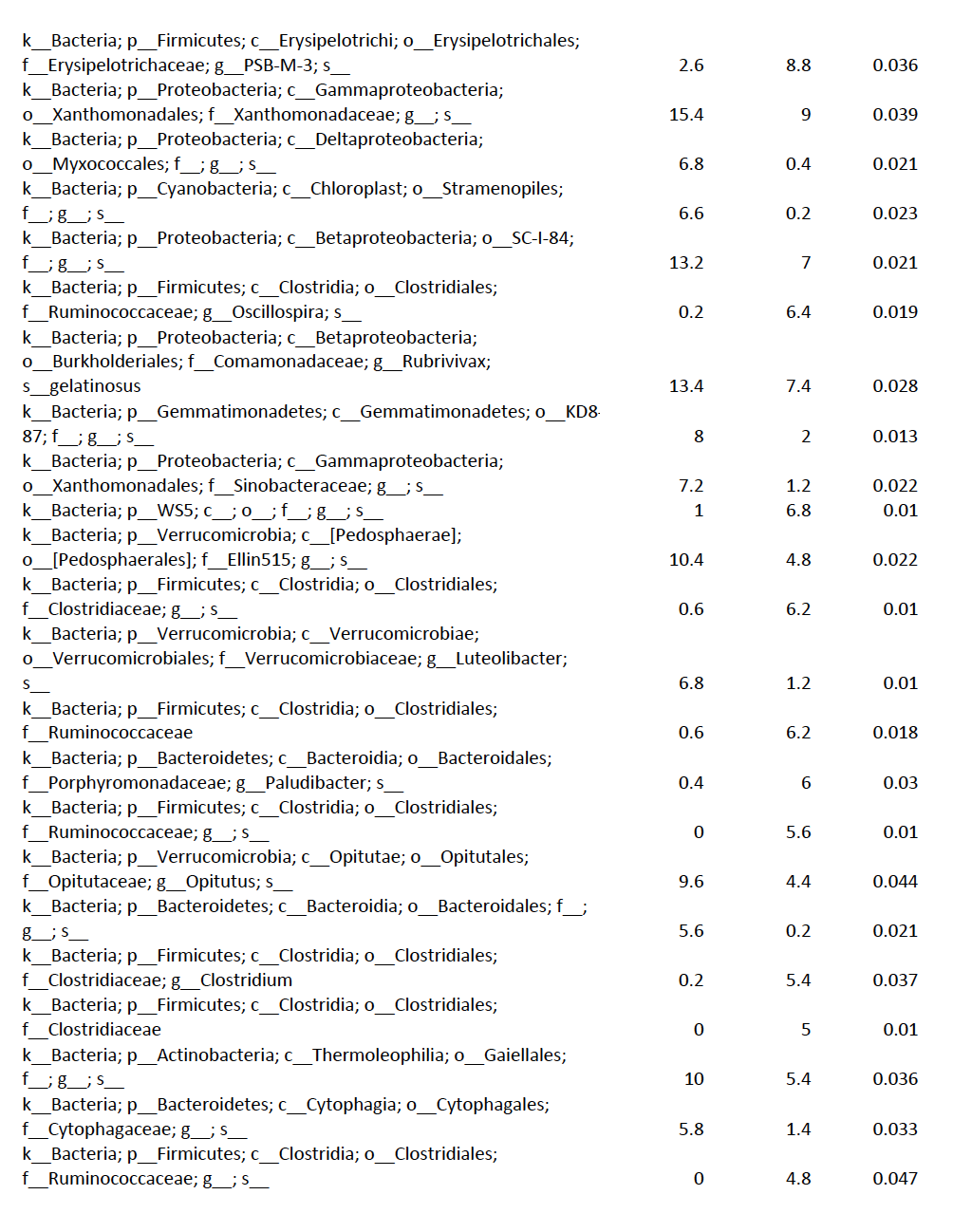

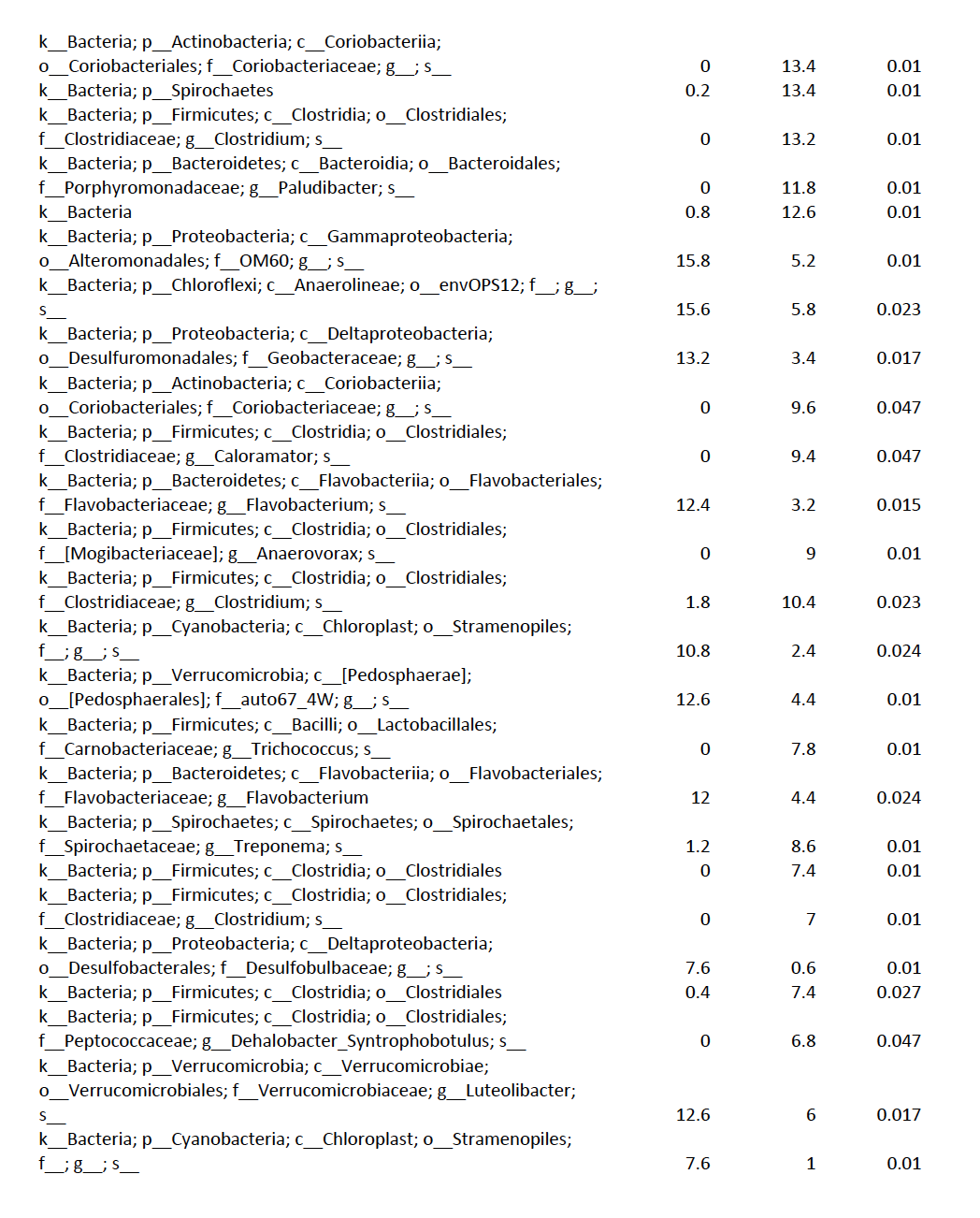

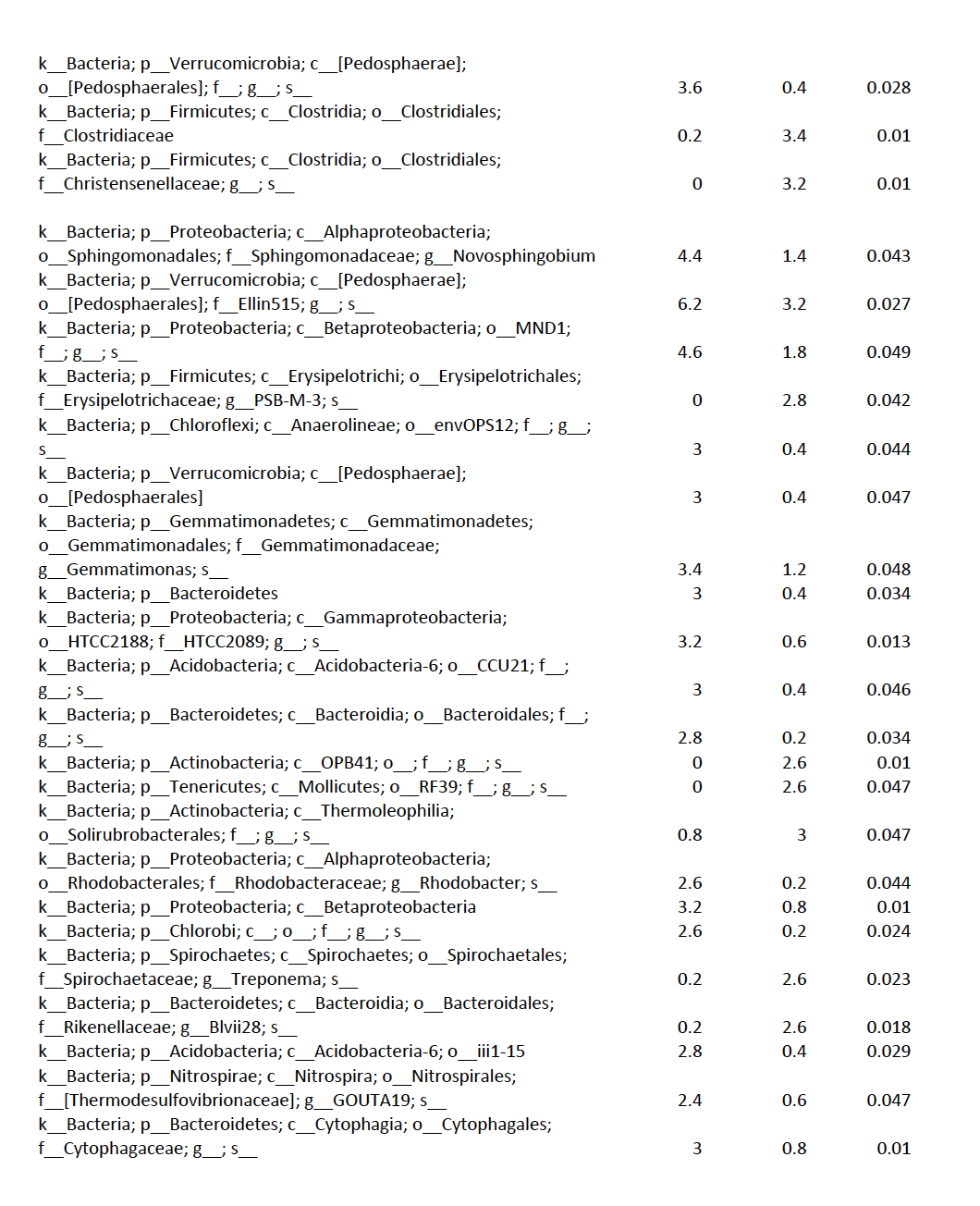

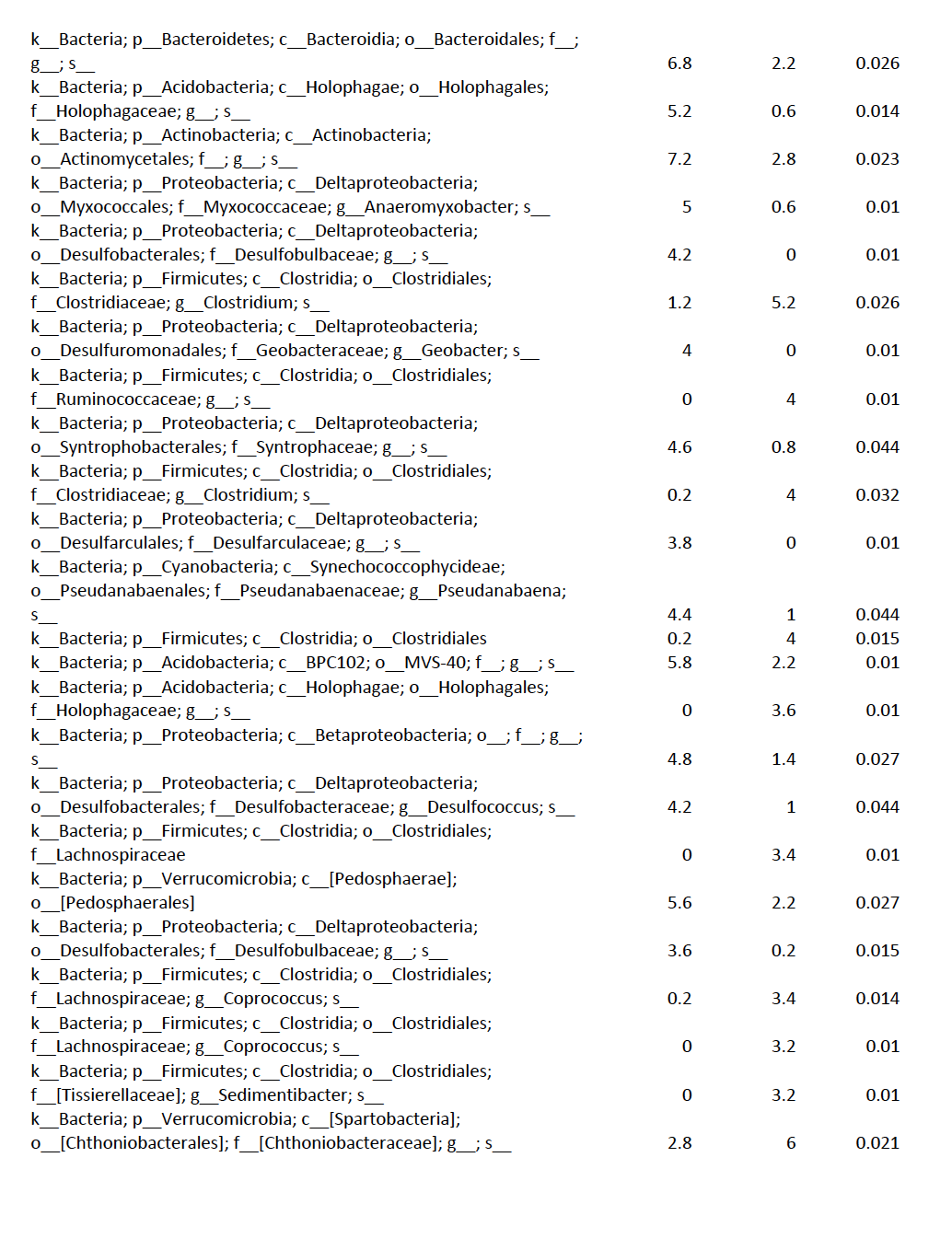

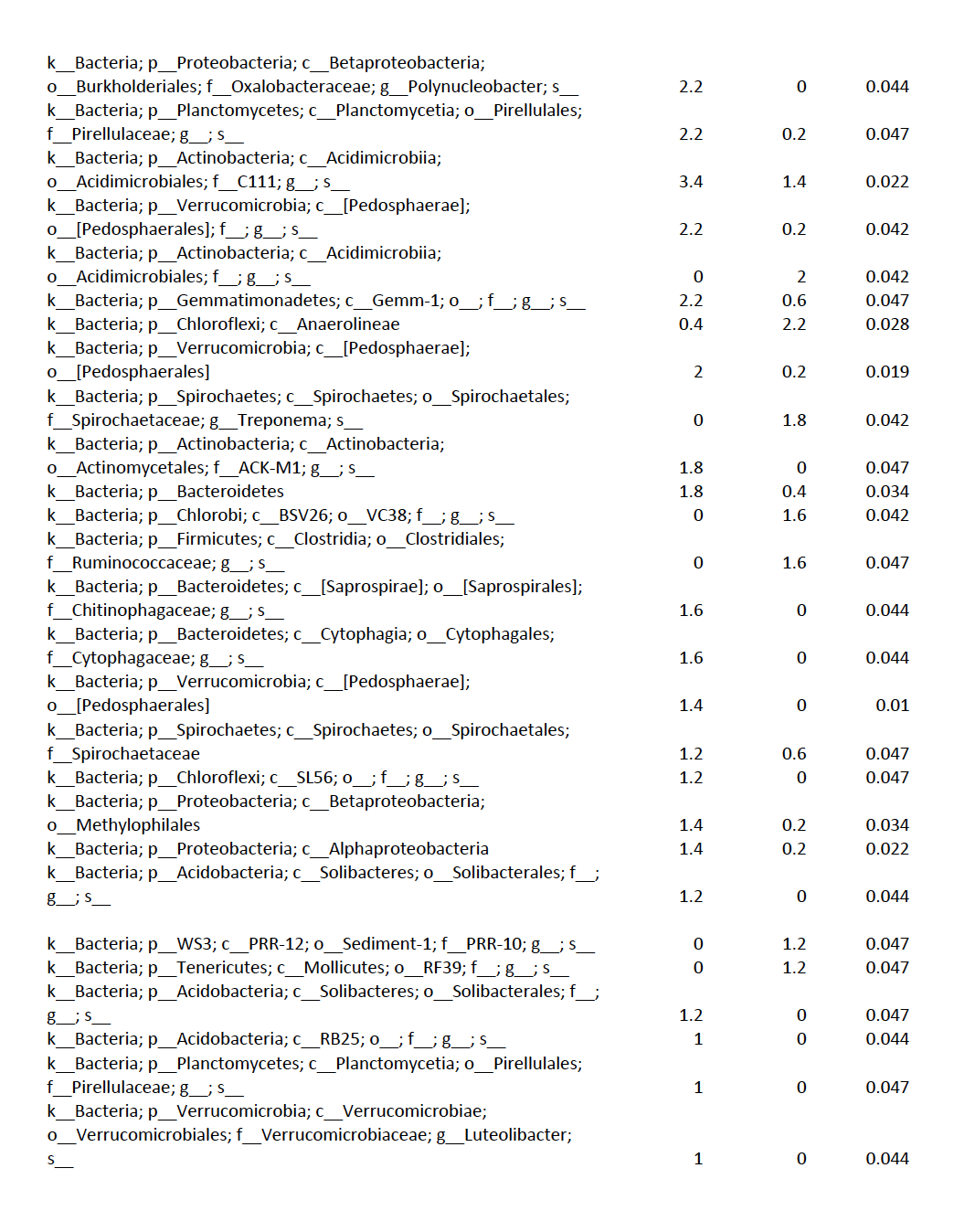

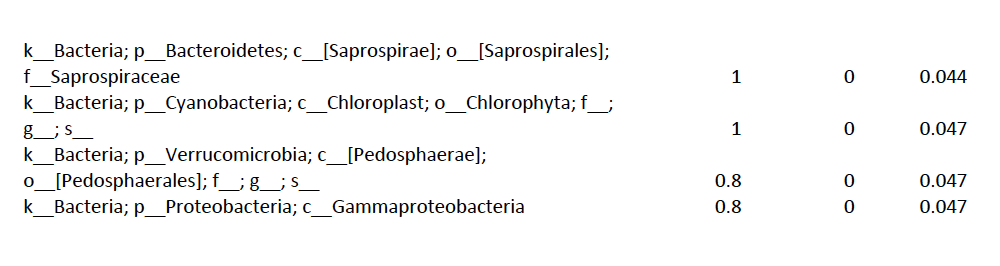


**Table S2.** Relative abundance of selected organisms. Abundance in ‘All OTUs’ is relative to the full community. Abundance in ‘Methylating OTUs’ is relative to methylating organisms only.
